# Supplementary figures and images for: Epithelial competition determines gene therapy potential to suppress Fanconi Anemia oral cancer risk
Source: PLoS Comput Biol. 2025 Sep 10;21(9):e1012915. doi: 10.1371/journal.pcbi.1012915 (PMC12453225; doi:10.1371/journal.pcbi.1012915)

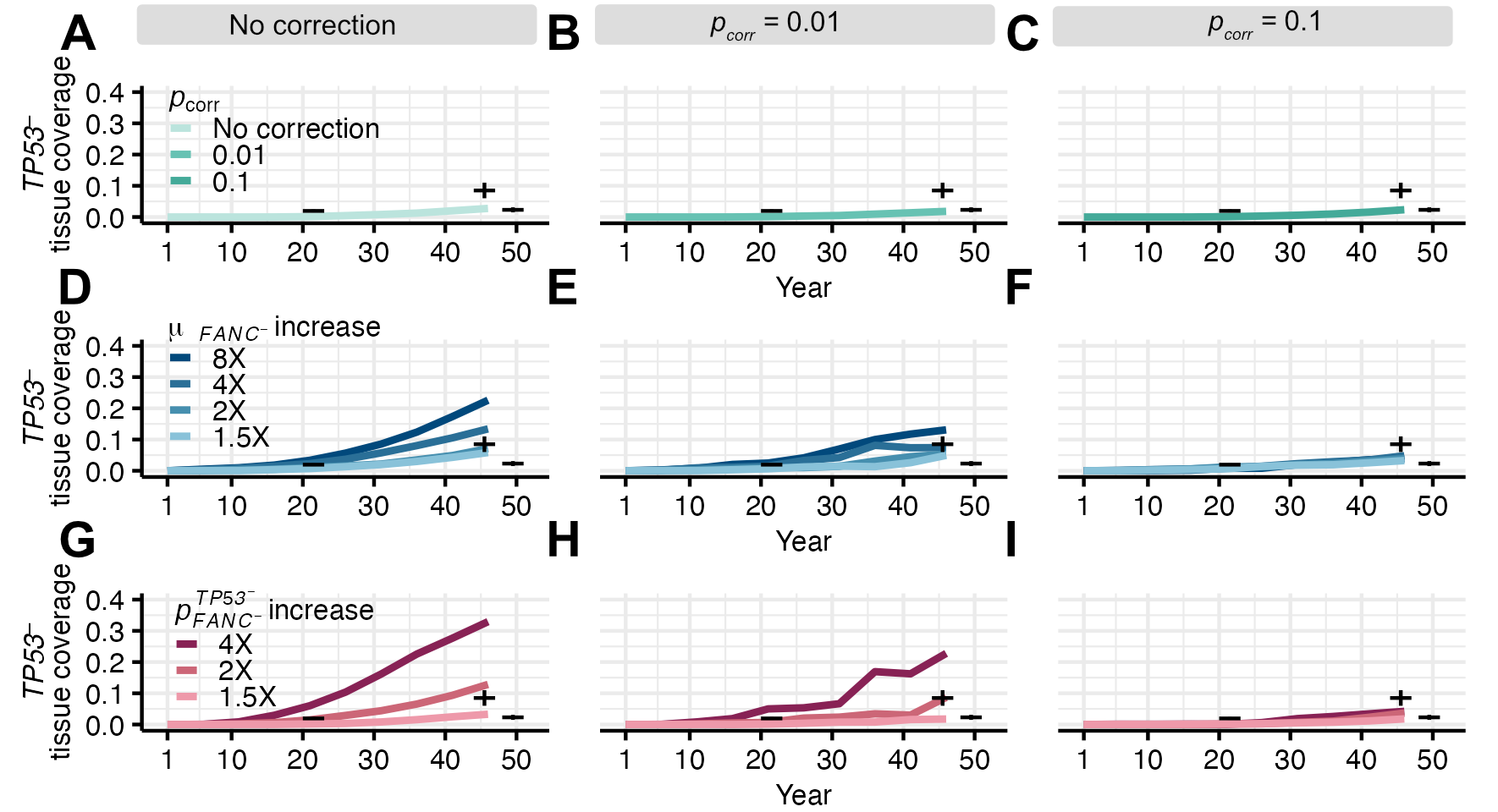

Supplement: S1 Fig — (A-I) TP53– tissue coverage tracked over time for 46 years on 0.33 mm2 simulated tissue sections with or without single gene correction (k = 30 cells and D = 2). Columns represent conditions with no correction, pcorr = 0.01 and pcorr = 0.1. Rows represent the experimental conditions as described in Fig 4. Black crosses represent TP53 tissue coverage ranges from Martincorena et al. [48] normal esophagus, with vertical lines indicating the observed range. 300 simulations were performed for each condition within each persistence coefficient for A-C and 100 simulations were performed for D-I. (TIF) [file pcbi.1012915.s001.tif]

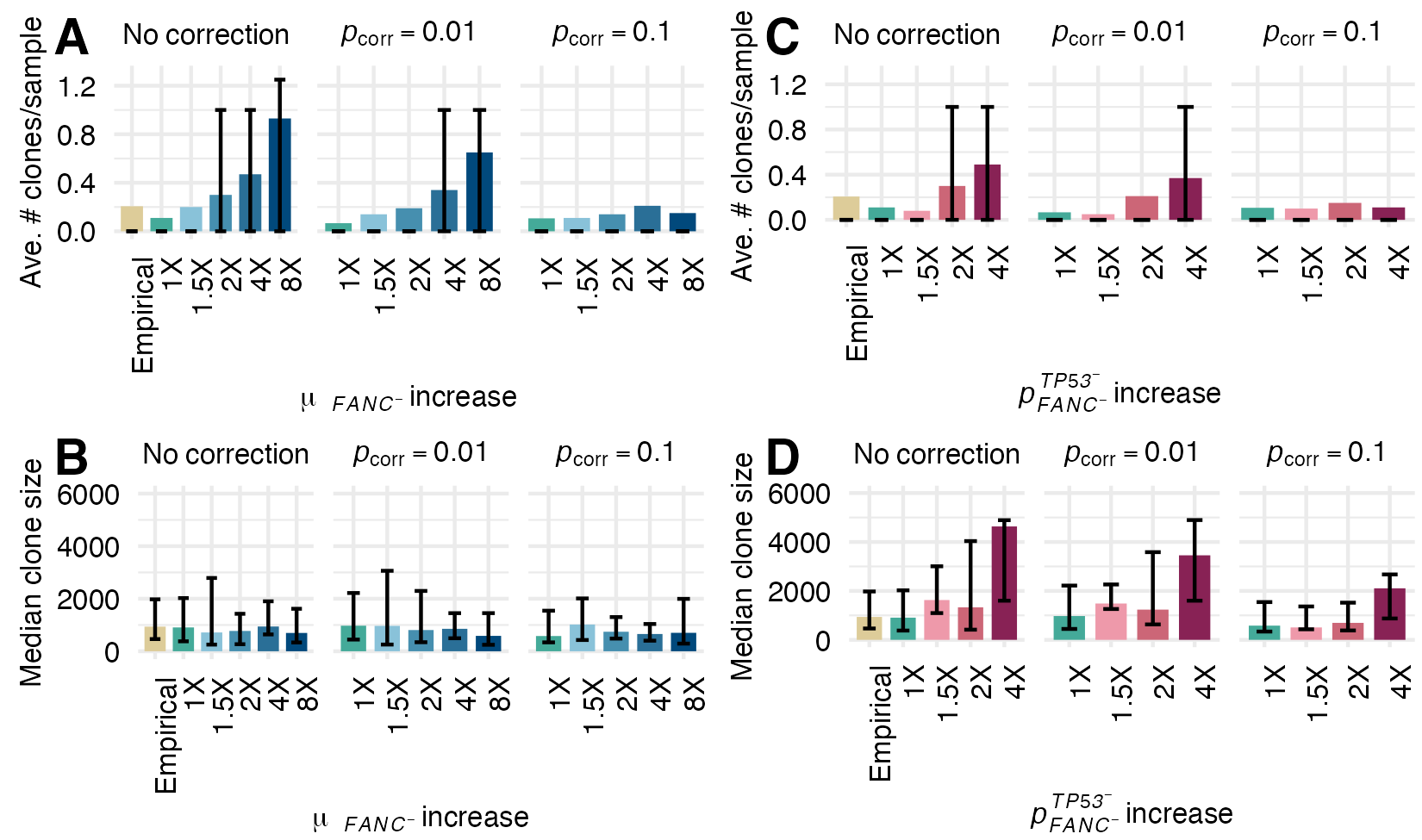

Supplement: S2 Fig — (A-D) The average number and size of TP53– clones after 46 years on 0.33 mm2 simulated tissue sections with (pcorr = 0.01 or 0.1) or without gene correction. The 1X condition in each panel (green) represents simulations where TP53 mutation rates and persistence coefficients are equal across FANC– and FANC+ cells. The empirical condition in each panel (yellow) represents data from downsampled normal esophageal tissue in ages 36-55 from Martincorena et al. [48]. 300 tissue simulations of the 1X condition and 100 tissue simulations of all other conditions were performed. Bars indicate interquartile ranges. (A) Average number of distinct TP53– clones when the TP53 mutation rate is elevated in FANC– cells (μFANC−=m·μFANC+, m=(1.5,2,4,8)). (B) Median TP53– clone size under the same conditions as (A). (C) Average number of distinct TP53– clones when TP53 persistence coefficients are elevated in FANC– cells (pFANC−TP53−=r·pFANC+TP53−,r=(1.5,2,4)). (D) Median TP53– clone size under the same conditions as (C). (TIF) [file pcbi.1012915.s002.tif]

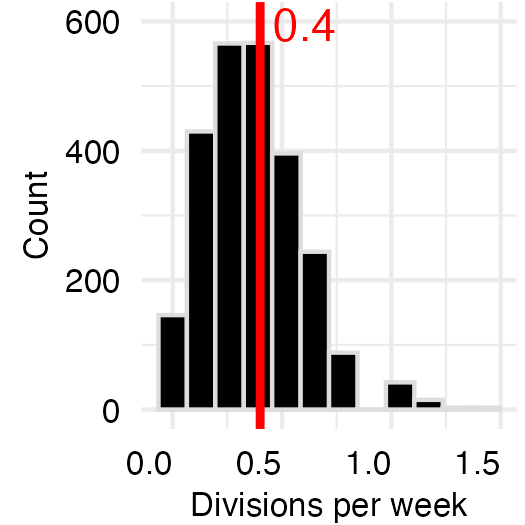

Supplement: S3 Fig — Distribution of average cell division rates across basal layer positions in the HomeostaticEpidermis model over 100 model timesteps on a 50x50 cell grid. The red line indicates the mean division rate (0.4 divisions per week). (TIF) [file pcbi.1012915.s003.tif]

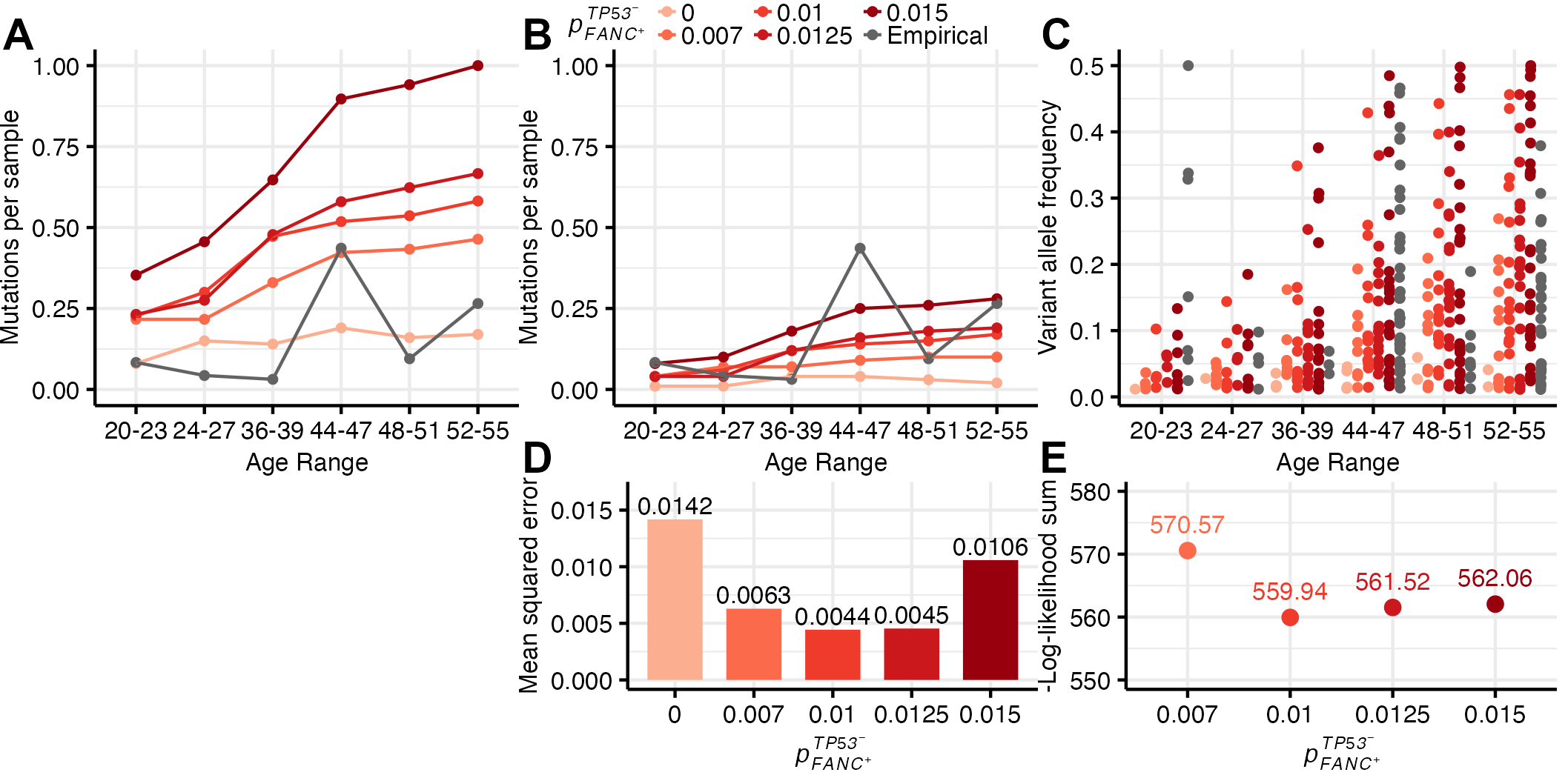

Supplement: S4 Fig — (A) The number of TP53 mutations per 0.33 mm2 tissue sample in simulations with varying persistence coefficients (pFANC+TP53−=0,0.007,0.01,0.0125,0.015) using the default mutation rate of the HomeostaticEpidermis model (colored by persistence coefficient), compared to empirical data (grey). 100 tissue simulations were performed for each age range and persistence coefficient. (B) The number of TP53 mutations per sample in simulations with varying persistence coefficients using the calibrated mutation rate (FANC+). (C) Variant allele frequencies of mutation meeting the lower limit of detection in simulated (colored by persistence coefficient) and empirical (grey) tissue. (D) Mean squared error between the simulated and empirical data for the number of TP53 mutations across various pFANC+TP53− values. Age range 44-47 was excluded from the mean squared error computation. (E) Negative log-likelihood comparisons of empirical variant allele frequencies against simulated distributions across pFANC+TP53− values. Age range 20-23 was excluded from negative log-likelihood computation. (TIF) [file pcbi.1012915.s004.tif]
